# Clinical Outcomes of Transepithelial Photorefractive Keratectomy Performed with Smart Pulse Technology for the Correction of Moderate to High Myopia

**DOI:** 10.3390/jcm13113058

**Published:** 2024-05-23

**Authors:** Tony Ho

**Affiliations:** Mount Elizabeth Medical Centre, Singapore 228510, Singapore; hokw888@me.com; Tel.: +65-(6733)-5188

**Keywords:** photorefractive keratectomy, transepithelial photorefractive keratectomy, smart pulse technology, Schwind AMARIS excimer laser, moderate to high myopia correction

## Abstract

**Purpose**: To evaluate the safety and efficacy of the transepithelial photorefractive keratectomy (TransPRK) performed using smart pulse technology (SPT) in myopic eyes with refractive error ranging from −5.25 D to −9.75 D. **Methods**: This retrospective study evaluated the outcomes of SPT-assisted TransPRK in 150 eyes performed using a 1050 Hz AMARIS excimer laser. **Results**: At 6 months postoperative, 98% of eyes achieved uncorrected distance visual acuity (UDVA) of 20/25 or better, and postoperative UDVA within one line of preoperative corrected distance visual acuity (CDVA). No eyes lost any line of CDVA. Residual spherical equivalent refraction and cylinder within ±0.50 D of intended correction were achieved in 72% and 67% of eyes, respectively. Ninety-seven percent of eyes reported no halos and glare. **Conclusions**: TransPRK using a 1050 Hz excimer laser with SPT showed excellent predictability, safety, and efficacy for moderate to high myopia correction.

## 1. Introduction

The conventional photorefractive keratectomy (PRK) procedure involves the manual or alcohol-assisted removal of corneal epithelium followed by excimer laser ablation to correct refractive errors. Manual or alcohol-assisted epithelial removal has some drawbacks, including prolonged epithelial healing secondary to basement membrane injury or potential alcohol toxicity, significant pain, delayed visual recovery, and variable degrees of stromal haze even with the use of mitomycin C [1]. Abnormal healing has been associated with under- or over-correction, refractive regression, etc. [2].

To overcome the drawbacks of conventional PRK, transepithelial photorefractive keratectomy (TransPRK) was initially introduced as a two-step procedure involving excimer laser-assisted phototherapeutic keratectomy (PTK) as a first step to remove the epithelium followed by stromal laser ablation as a second step [3,4], assuming the uniform thickness of epithelium across the ablation zone [5].

A series of advancements have been made to the TransPRK procedure. Excimer laser-assisted removal of the epithelium and the stroma is now performed as a single-step procedure that typically ablates 55 μm of central tissue and about 65 μm of peripheral tissue (thus removing the epithelium) for an 8 mm ablation zone [6]. Due to the high-frequency laser and one-step procedure, the risk of dehydration is mitigated.

Smart pulse technology (SPT) is a new software feature of AMARIS’ excimer laser platform to further diminish the postoperative surface irregularities of the residual stromal bed at the end of the treatment. The reported clinical benefits of SPT-assisted surface ablation are smoother cornea, faster corneal re-epithelialization, less pain and discomfort, earlier postoperative visual recovery, and less haze occurrence than surface ablation without SPT [7].

Schwind AMARIS 1050 Hz excimer laser platform uses a reverse single-step TransPRK procedure, in which ablation to correct the refractive error is performed first, followed by ablation to simulate the epithelium thickness profile of the normal population [8]. This also maximizes the smoothing effect since it has been recognized that epithelial thickening occurs in areas of stromal irregularity. This potential advantage is negated when the epithelium is removed before stromal ablation, thus exposing the underlying irregularity [9].

The present study aimed to retrospectively examine the safety and efficacy of the SPT-assisted TransPRK procedure in myopic eyes with refractive error ranging from −5.25 D to −9.75 D.

## 2. Materials and Methods

### 2.1. Study Population

This retrospective chart review included 150 eyes of 75 patients with or without astigmatism who had undergone SPT-assisted TransPRK with AMARIS 1050 Hz excimer laser (SCHWIND eye-tech-solutions, GmbH, Kleinostheim, Germany) using the all surface laser ablation algorithm for the correction of myopia (−5.25 D to −9.75 D) between September 2021 and March 2022 at Clear Vision Eye Clinic and LASIK Centre, Singapore, and for whom the postoperative 6 month data were available. The present study did not include case records of patients who underwent TransPRK for amblyopia correction.

All surgeries were performed by an experienced surgeon (TH). The study followed the tenets of the Declaration of Helsinki. Data were collected as a part of routine clinical practice, and only the de-identified and anonymized patient data were used in the study. Owing to the retrospective nature of the study, ethics approval was not required.

As a routine clinical practice, laser vision correction (LVC) is performed on patients who are 18 years or older, have stable refractive error for at least 12 months, have normal keratometry (K) readings, normal topography, and an estimated residual bed thickness of at least 350 µm. Patients were asked to discontinue soft and hard contact lenses 3 days and 2 weeks (respectively) before the LVC workup. Patients with keratoconus or those at risk of developing post-LASIK ectasia, systemic connective tissue diseases that might influence corneal healing, were not considered for surgery.

### 2.2. Preoperative Examination

The preoperative examination included assessment of uncorrected distance visual acuity (UDVA), corrected distance visual acuity (CDVA), intraocular pressure (IOP), cycloplegic and manifest refraction, slit lamp biomicroscopic examination, fundus evaluation, corneal topography/tomography (Orbscan, Bausch & Lomb Incorporated, Bridgewater, NJ, USA; Sirius, CSO, Florence, Italy; Pentacam, Oculus, Arlington, WA, USA), and corneal pachymetry (Lenstar, Haag Streit, Köniz, Switzerland and IOLMaster 700, Carl Zeiss Meditec AG, Jena, Germany).

### 2.3. Surgical Technique

All patients underwent SPT-assisted TransPRK with AMARIS 1050 Hz excimer laser (SCHWIND eye-tech solutions, Kleinostheim, Germany). The TransPRK software (Version 6.1.2117) performed an epithelial ablation depth of 55 µm in the center that increased towards the periphery in a parabolic pattern (56 µm at 2.0 mm diameter, 58 µm at 4.0 mm, 61 µm at 6.0 mm, and 65 µm at 8.0 mm). The ablation profile was centered on the corneal vertex (determined using the topography), taking 100% of the pupil offset value and closely approximating the visual axis. A high-speed eye tracker with an acquisition speed of 1050 Hz that simultaneously tracks both the limbus and the pupil with a reaction time of less than 3 ms was used. Due to the predictive algorithm of AMARIS 1050 RS, eye movements occurring during the period between the acquisition of the eye-tracker image and triggering of the subsequent laser pulses are anticipated and pre-compensated, which results in zero latency for the laser system.

The target refraction was +0.25 D in all the cases. For astigmatism correction, the surgeon relied on the actual keratometric diopter power and the power axis. If the keratometric and manifest cylinder differed in magnitude and axis, the surgeon adjusted the magnitude and axis between the two values and treated them accordingly. After the ablation, 0.02% mitomycin C was applied to the stromal bed for 15 s and rinsed with cold BSS (balanced salt solution). Following TransPRK, accelerated collagen crosslinking (CXL) treatment was performed in myopic eyes requiring spherical correction of more than 8 D, or eyes that had an estimated residual corneal bed thickness of 360 µm or less, using the KXL^®^ System (Avedro, Inc., Waltham, MA, USA) as a preventive measure against the development of iatrogenic keratectasia. The soaking time with riboflavin 0.1% solution in HPMC was 60 s, followed by UV irradiation at 2.7 J/cm^2^ (30 mW for 60 s) [10]. A bandage contact lens was applied until the epithelium healed completely. A 15 mL bottle of Balanced salt solution (BSS) was prescribed to be used as lubricant eye drops during the period that bandage contact lenses were worn and replaced with standard over-the-counter (OTC) preservative-free eyedrops only after the bandage lenses were removed.

Patients were asked to strictly adhere to the standard postoperative medication regimen that included tobramycin and dexamethasone ophthalmic suspension (Tobradex, Alcon), moxifloxacin (Vigamox, Alcon, Geneva, Switzerland), and ketorolac ophthalmic Solution (Acular, Allergan, Dublin, Ireland). Acular NSAID (non-steroidal anti-inflammatory drug) eye drops were given only for the first two days.

Pred Forte eyedrops were used at more frequent doses for the first two weeks and tapered down to three times a day by the fourth week and then switched to less potent fluorinated eyedrops such as loteprednol (Lotemax, Bausch & Lomb, Rochester, NY, USA) for the second and third month. Antibiotic eyedrops were discontinued after one week, and steroid eye drops were discontinued after three months. During the three months of steroid use, patients were frequently monitored for intraocular pressures (IOPs) at regular follow-ups. Steroid responders were put on less intensive steroid eyedrops and followed up more frequently and were also given prophylactic IOP-reducing drops such as timolol and brimonidine. Patients were also advised to avoid exposure to direct sunlight, and wear polarized wraparound sunglasses for one month after the surgery whenever outdoors in the sun.

### 2.4. Study Parameters

The safety and efficacy parameters analyzed at 6 months postoperative included assessment of uncorrected and corrected visual acuity (UDVA, CDVA), intraocular pressure (IOP), safety index (defined as the ratio of postoperative and preoperative decimal CDVA), efficacy index (defined as the ratio of postoperative decimal UDVA to preoperative decimal CDVA), spherical equivalent refraction, refractive astigmatism, and corneal haze assessed on a scale of 0 to 4, 0: no haze (clear corneas), 1: trace haze, 2: mild haze, 3: moderate haze and 4: severe haze.

Postoperative complications and patient-reported outcomes such as glare/halos (graded on a scale of 0 to 3 where 0: no halo/glare, 1: mild, 2: moderate, 3: severe) and dry eye symptoms (graded on a scale of 0 to 3 where 0: no dry eye, 1: mild, 2: moderate and 3: severe dry eye) were also recorded.

### 2.5. Statistical Analysis

Descriptive statistics, including mean and standard deviation, were used to represent the data of continuous variables, and for categorical variables, the results were expressed as numbers and percentages. Standard graphs were used to report refractive surgery outcomes.

## 3. Results

A total of 150 eyes of 75 patients with myopia ranging from −5.25 D to −9.75 D were included in the present study. The mean age of the study participants was 27.2 ± 3.6 years (range 20 to 34).

### 3.1. Visual Outcomes

**Figure 1:**
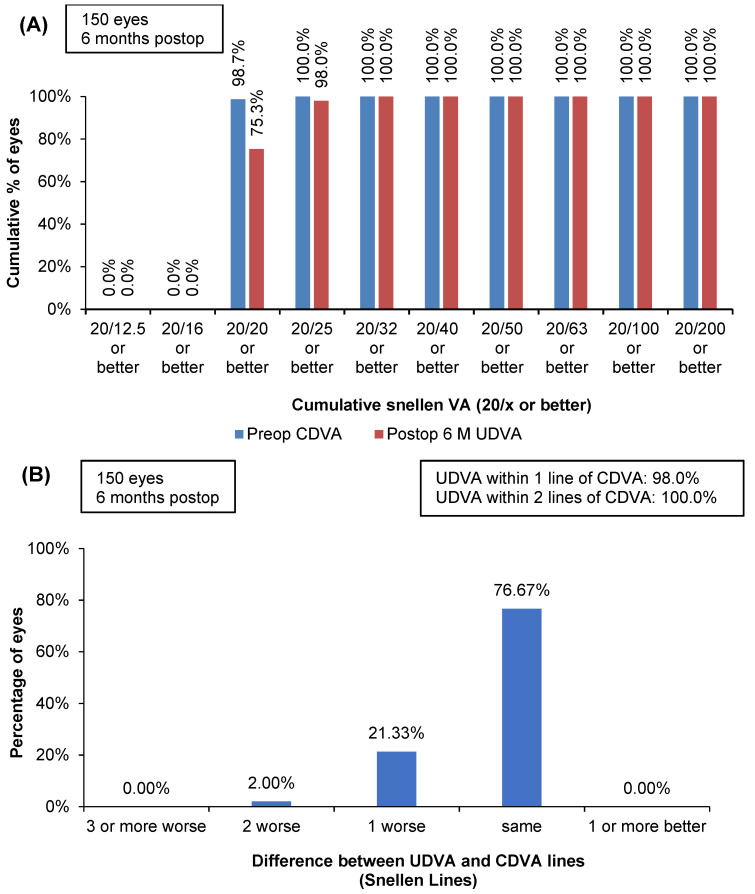

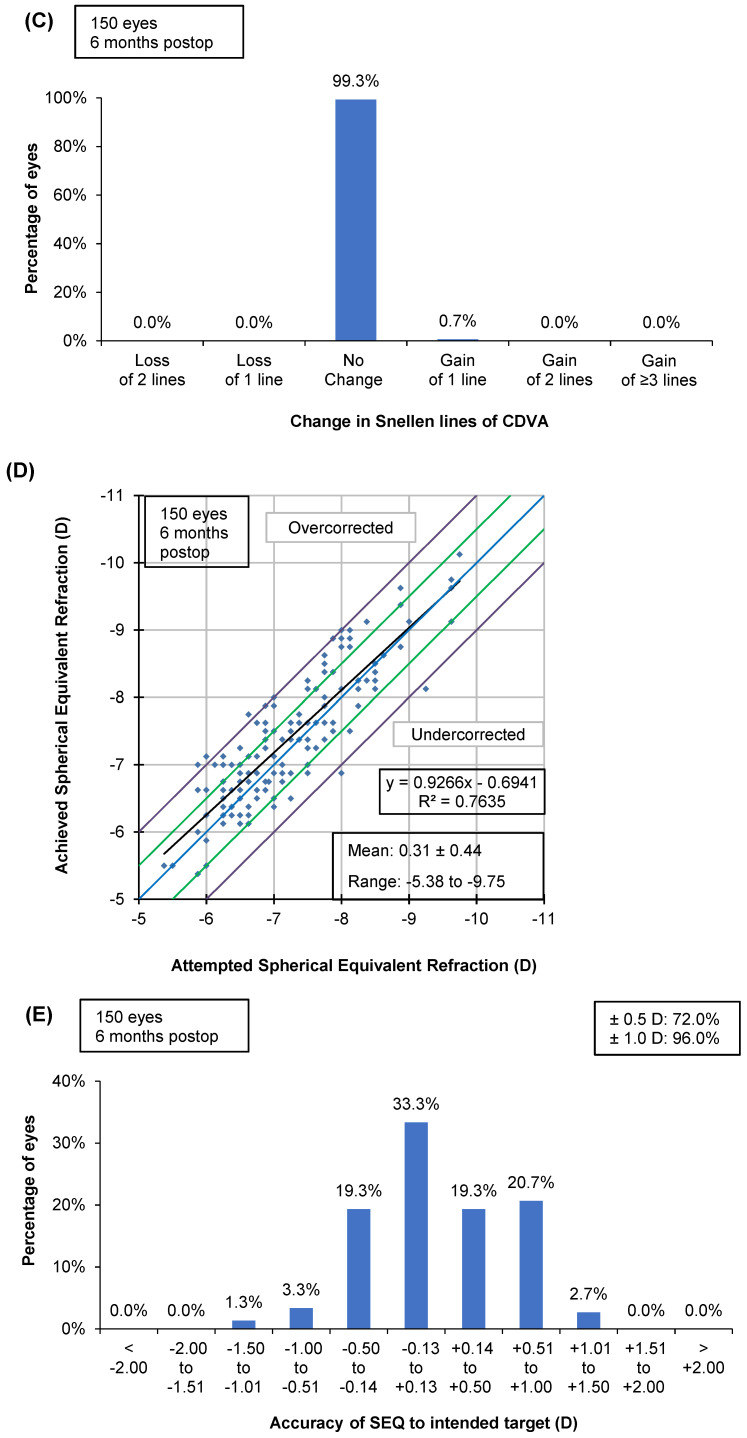

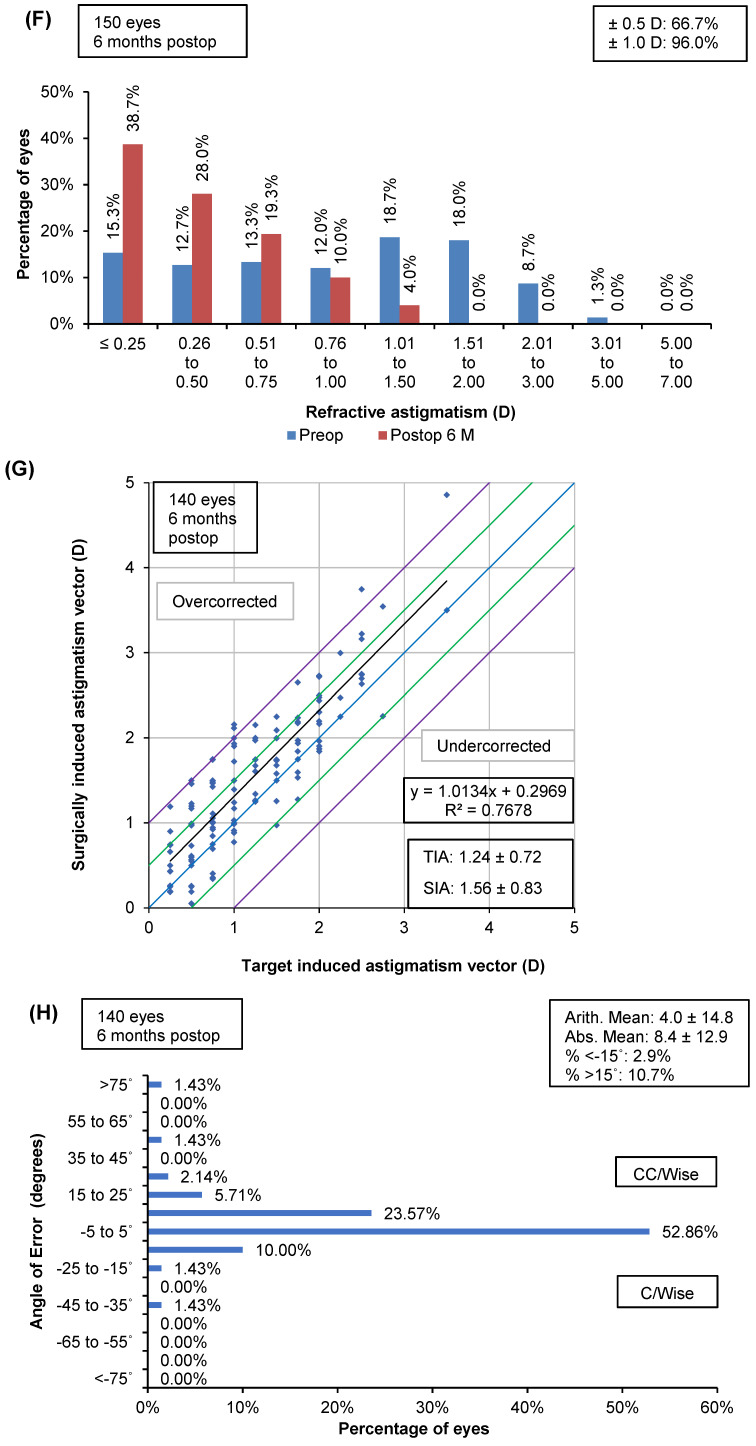
Outcomes of transepithelial advanced surface ablation conducted with the 1050 Hz excimer laser coupled with the smart pulse technology ablation software (Version 6.1.2117) at six months postoperative. (**A**) Efficacy chart depicting cumulative 6-month postoperative UDVA and preoperative CDVA; (**B**) Difference between uncorrected distance visual acuity postoperatively and corrected distance visual acuity preoperatively; (**C**) Change in Snellen lines of CDVA from preop to 6 months postop; (**D**) Scatterplot of Attempted versus achieved spherical equivalent refraction (square boxes represent the attempted versus achieved spherical equivalent refraction of each eye). The blue line indicates that achieved refraction is equal to attempted refraction. Green lines indicate eyes within ±0.5 D of attempted refraction, and purple lines indicate eyes within ±1.00 D of attempted refraction; (**E**) Accuracy of spherical equivalent refraction to the intended target; (**F**) Refractive astigmatic accuracy; (**G**) Scatterplot of TIA (target-induced astigmatism) versus SIA (surgically-induced astigmatism) (square boxes represent the TIA versus SIA of each eye). The blue line indicates TIA is equal to SIA. Green lines indicate eyes within ±0.5 D of TIA, and purple lines indicate eyes within ±1.00 D of TIA; (**H**) Angle of error graph.

Of the 150 eyes, 98% of eyes achieved a UDVA of 20/25 or better, and postoperative UDVA was within one line of preoperative CDVA in 98% of the eyes at postoperative 6 months (Figure 1A,B). As many as 99.3% of the eyes showed no change in Snellen lines of CDVA, and 0.7% showed a gain of one line of CDVA at 6 months postoperative. None of the eyes lost any lines of CDVA postoperatively (Figure 1C). The efficacy index was 1.1, and the safety index was 0.99.

### 3.2. Refractive and Astigmatic Outcomes

Mean MRSE reduced significantly from −7.08 ± 0.95 D preoperatively to 0.31 ± 0.44 D at 6 months postoperative. Figure 1D shows the scatterplot between the attempted and achieved spherical equivalent refractive correction. Figure 1E shows the predictability of refractive correction; 72% and 96% of eyes achieved postoperative residual spherical equivalent refraction within 0.50 D and 1.00 D of intended correction, respectively. At the 6-month follow-up, 67% and 96% of eyes had residual cylinder within 0.50 D and 1.00 D, respectively (Figure 1F).

Figure 1G plots TIA (target-induced astigmatism) against SIA (surgically-induced astigmatism) on 140 astigmatic eyes targeted for emmetropia.

### 3.3. Corneal Haze

**Figure 2:**
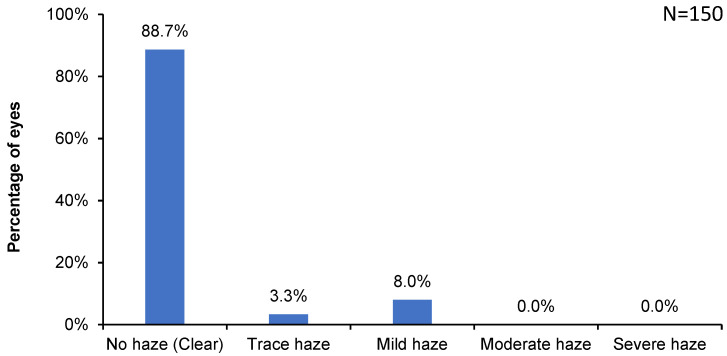
Incidence of corneal haze at 6 months postoperative.

Assessment of postoperative corneal clarity at 6 months revealed clear corneas (no haze formation) in 89% of eyes (Figure 2, Table 1). In 11.3% of eyes, a trace to mild levels of corneal haze were seen.

### 3.4. Patient-Reported Outcomes

**Figure 3:**
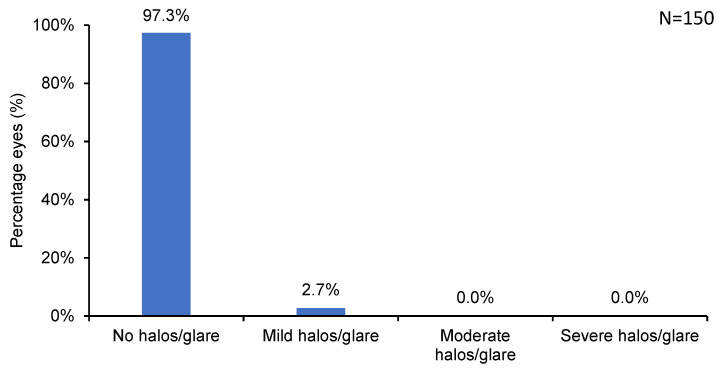
Incidence of halos/glare at 6 months postoperative.

**Figure 4:**
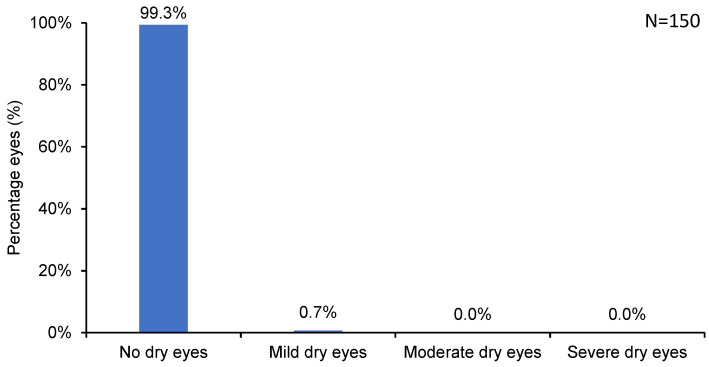
Incidence of postoperative dry eye.

As many as 97.3% of eyes reported no halos and glare, and 2.7% reported only mild ones (Figure 3, Table 1) at 6 months postoperative. Essentially all (99.3%) eyes reported no dry eye (0.7% of eyes had mild dryness) postoperatively (Figure 4, Table 1).

### 3.5. Intraocular Pressure (IOP)

The mean IOP was 18.33 ± 2.25 mmHg at baseline and 12.42 ± 1.87 mmHg at 6 months postoperative.

### 3.6. Adverse Events

None of the patients reported any adverse events during the study.

## 4. Discussion

The introduction of SPT-assisted TransPRK has made surface ablation procedures relatively faster, with less postoperative pain, quicker re-epithelization, and faster visual recovery [11]. While the reported clinical outcomes of TransPRK in myopic eyes are generally excellent [12,13,14], there is a need for more data on SPT-assisted TransPRK in high myopia eyes [7].

In the present study, TransPRK with a 1050 Hz excimer laser was implemented using SPT in 150 eyes with myopia of −5.25 D to −9.75 D, which resulted in excellent visual and refractive outcomes. At 6 months postoperative, 98% of eyes achieved UDVA of 20/25 or better. None of the eyes lost any line of CDVA. A residual refractive cylinder within 0.50 D and 1.00 D of intended correction was seen in 82% and 94.1% of eyes, respectively. Improved visual acuity outcomes achieved in the present study are in agreement with the previously published literature that also reported excellent postoperative uncorrected and corrected distance visual acuity outcomes following SPT-assisted surface ablation [7,15].

TransPRK performed with the high speed 1050 Hz excimer laser shortens treatment time and minimizes the risk of corneal dehydration. This no-touch procedure removes the epithelium without suction, flap, or blade, and without using alcohol, and the entire procedure is completed in a single step. The TransPRK procedure evaluated in this study ablates the refractive component first, followed by the ablation of the epithelial profile. This reverse sequence utilizes the early period of better patient cooperation and fixation for the critical refractive ablation, leaving less alignment-critical epithelial ablation for the end. As the size of the epithelial removal zone matches the size of the ablation zone, the TransPRK procedure is associated with smaller epithelial defects and faster re-epithelialization [16].

Corneal haze formation is one of the common complications of surface laser ablation techniques such as PRK [17,18]. The risk of corneal haze following PRK increases with increasing correction and volume of stromal tissue removal [19,20,21]. However, in the present study involving moderate to high myopia eyes, 89% of the eyes had clear corneas, and only 11% of the eyes showed trace (3%) to mild (8%) haze formation at the 6-month follow-up. This lower incidence of haze observed in the present study involving moderate to high myopia eyes could be attributed to the use of SPT-assisted TransPRK and mitomycin C. The SPT-assisted laser ablation with Schwind AMARIS laser delivers laser spots based on a curved corneal surface using a three-dimensional fullerene structure, meaning that each ablation point is equidistant [22]. The latest measurement and analysis methods also help optimize the ablative spot geometry, which avoids thermal load, resulting in a smooth corneal surface and a smaller, healthier, neater epithelial edge. A smoother corneal stromal bed resulting from SPT-assisted TransPRK has been associated with faster corneal re-epithelialization, earlier postoperative visual recovery, a shorter period of patient discomfort [23], and less haze occurrence than surface ablation without SPT [24].

In the present study, 70% of the eyes underwent TransPRK combined with accelerated corneal collagen crosslinking (CXL treatment). This was carried out to enhance strength, stabilize the residual stromal bed, and minimize the risk of iatrogenic ectasia. The use of dextran-free HPMC riboflavin solution in the present study potentially resulted in a better crosslinking effect and little impact on corneal thickness [25,26]. PRK combined with CXL is known to cause apoptosis of keratocytes in the anterior stroma [27]. The lower incidence of corneal haze in the present study might also be attributed to the combined CXL treatment that induces apoptosis of keratocytes, thereby inhibiting the production of haze-causing extracellular substances by keratocytes [27].

Surface ablation procedures are associated with a lesser incidence of dry eye [28]. In the present study, only 0.7% of the eyes showed mild dry eye symptoms, and 99.3% experienced no dry eyes postoperatively. Furthermore, there were no complaints of postoperative glare/halos in 97.3% of our patient population, and less than 3% experienced mild glare/halos. Factors such as smooth residual stromal bed achieved using AMARIS 1050 Hz laser with SPT, and the relatively larger optical zone diameters (range 6.0 to 6.7 mm) achievable with the tissue-sparing surface ablation method might have contributed to the lower incidence of visual phenomena [15,29]. In the present study, the calculated residual stromal bed thickness was maintained above 350 µm for all patients. Other contributing factors may include copious use of BSS as lubricating eye drops during the first five days of epithelial healing when the bandage contact lens was worn, stringent counseling on avoidance of UV rays during the healing process, and intensive but careful use of the aforementioned anti-inflammatory drug regimen.

In the author’s experience, using BSS lubrication for the first five days postoperatively when bandage contact lenses are worn is very effective in relieving eye pain or discomfort and complaints of blurry vision compared to the standard OTC preservative-free lubricating eyedrops containing castor oil, hypromellose, or hyaluronidase. Furthermore, using Acular NSAID eye drops for the first two days effectively reduces eye puffiness, redness, and tearing.

Correcting refractive errors using surface ablation is typically associated with intense postoperative pain, more extended periods of wound healing, etc. Improvements in surface ablation techniques such as SPT-assisted TransPRK, the use of the author’s customized postoperative regimen of anti-inflammatory medications, and lubricating eye drops have made surface ablation procedures comfortable with less postoperative pain, faster wound healing, and visual recovery. Quicker patient recovery translates into greater treatment acceptance as patients need to take less time off work. In the author’s opinion, this no-touch TransPRK procedure is comfortable. It can be used safely and effectively to correct refractive errors even in patients with thin corneas, high refractive power, or sports personnel engaged in contact sports where other LVC methods are contraindicated.

No adverse events were reported during the study period. The mean postoperative IOP was reduced (compared to baseline), which might be attributed to the relative flattening and thinning of the central cornea and the absence of Bowman’s membrane following TransPRK [30,31].

The present study is limited by its retrospective design and the shorter follow-up time of 6 months. Owing to the retrospective study design, information about the individual effect of the 1050 Hz excimer laser and SPT could not be ascertained. Future prospective comparative studies with larger sample sizes and long term follow ups may provide more robust data related to the safety and efficacy of SPT-assisted TransPRK. Nevertheless, the results are meaningful to the refractive surgeons concerning refractive correction in patients with moderate to high myopia.

## 5. Conclusions

To conclude, TransPRK with the 1050 Hz excimer laser using the SPT treatment algorithm showed excellent predictability, safety, and efficacy for correcting moderate to high myopia.

## Figures and Tables

**Table 1:** Incidence of postoperative complications following SPT-assisted TransPRK.

	No	Trace	Mild	Moderate	Severe
**Haze**	88.7%	3.3%	8.0%	0.0%	0.0%
**Halos/Glare**	97.3%	2.7%	0.0%	0.0%	0.0%
**Dry eye**	99.3%	0.7%	0.0%	0.0%	0.0%

## Data Availability

Data underlying the results presented in this paper are not publicly available at this time but may be obtained from the authors upon reasonable request.
